# Modeling the Contribution of Milk to Global Nutrition

**DOI:** 10.3389/fnut.2021.716100

**Published:** 2022-01-13

**Authors:** Nick W. Smith, Andrew J. Fletcher, Jeremy P. Hill, Warren C. McNabb

**Affiliations:** ^1^Riddet Institute, Massey University, Palmerston North, New Zealand; ^2^Sustainable Nutrition Initiative, Riddet Institute, Massey University, Palmerston North, New Zealand; ^3^Fonterra Research and Development Centre, Palmerston North, New Zealand

**Keywords:** sustainable food systems, mathematical modeling, food production, nutrient requirements, population nutrition, milk

## Abstract

Nutrient-rich foods play a major role in countering the challenges of nourishing an increasing global population. Milk is a source of high-quality protein and bioavailable amino acids, several vitamins, and minerals such as calcium. We used the DELTA Model, which calculates the delivery of nutrition from global food production scenarios, to examine the role of milk in global nutrition. Of the 29 nutrients considered by the model, milk contributes to the global availability of 28. Milk is the main contributing food item for calcium (49% of global nutrient availability), Vitamin B2 (24%), lysine (18%), and dietary fat (15%), and contributes more than 10% of global nutrient availability for a further five indispensable amino acids, protein, vitamins A, B5, and B12, phosphorous, and potassium. Despite these high contributions to individual nutrients, milk is responsible for only 7% of food energy availability, indicating a valuable contribution to global nutrition without necessitating high concomitant energy intakes. Among the 98 food items considered by the model, milk ranks in the top five contributors to 23 of the 29 nutrients modeled. This quantification of the importance of milk to global nutrition in the current global food system demonstrates the need for the high valuation of this food when considering future changes to the system.

## Introduction

Achieving global nutrition and food security is a great challenge given the large forecast increases in the global population and the current degree of undernutrition, overnutrition, and inequitable food distribution present in many parts of the world (1–3). Nutrient-rich foods will be one of the solutions important in ensuring that the global population is nourished. Moreover, foods with high nutrient to energy ratios will be important in achieving nutrition without the attendant non-communicable disease risks and healthcare costs of excess energy intake (3).

Milk and its products are well-established as foods with high nutrient content and many links to positive health outcomes, with few links to negative outcomes (4–6). Mammalian milks are consumed throughout the lifespan and in all parts of the world, with global milk production for human consumption in excess of 800 million tons per year and growing (7, 8). Milk and other dairy food products produced from further processing of milk are thus a large contributor to human nutrition.

We know the production patterns of milk globally and in different parts of the world: 81% of 2018 milk production was cow's milk, with 15% from buffalo and smaller contributions from other mammals, such as goats, sheep, and camels (9). Moreover, decades of research have established the nutritional content of many mammalian milks consumed by humans. However, there is a lack of knowledge unifying these data to understand how these nutrients contribute to human nutrition as part of the broader global food system. We used the DELTA Model (10), a tool for investigating the nutrient availability supplied by the global food system, to analyze the contribution of mammalian livestock milk production to global nutrition. The model allows us to see the portion of nutrients supplied by milk, and to compare the relative contribution between milk and other food types.

## Materials and Methods

The DELTA Model was developed using data from publicly available data sources alongside biological values from the scientific literature and constructed in R (version 4.0.2). The results in this article were generated using version 1.2 of the model, the full mechanism of which is detailed in a previous publication (10). The model itself is available online.^1^ A brief description of the model structure is provided here.

The main data source for the DELTA Model is the Food and Agriculture Organization (FAO) Food Balance Sheets (FBS). These record production quantities, feed and food use, supply chain losses, and processing chains for 98 food commodities from 1961 to 2018. The DELTA Model takes a linear interpolation of the most recent 20 years of this data to represent the 2018 global food system, and we refer to this data throughout as the baseline dataset. In addition, the DELTA Model uses a more detailed set of foods than the FBS by subdividing the FBS commodities into a total of 315 food types, with relative quantities, processing yields, and inedible portions for each, derived either from FAO or scientific literature sources. Using this data, the DELTA Model calculates the total amount of each food type available for human consumption per year. From this value is deducted an in-home waste fraction, based on FAO estimates for each food group in different global regions (11).

Food composition data from the United States Department of Agriculture (12) is used to convert the available quantity of each food type into an available quantity of 29 essential nutrients (see Table 1 for a list of nutrients).

**Table 1:** Contribution of milk production to 2018 global nutrient availability.

	**Nutrient**	**Percentage of global availability provided by milk nutrition**	Ranked nutrient contribution position of milk nutrition^*^	Ranked nutrient density position of milk nutrition^*^
**Macronutrients**	Carbohydrates	3	5^th^	54^th^
	Energy	7	3^rd^	71^st^
	Fat	15	1^st^	38^th^
	Fiber	0	No contribution	No contribution
	Protein	12	3^rd^	41^st^
**Micronutrients**	Calcium	49	1^st^	8^th^
	Copper	2	9^th^	69^th^
	Folate	3	6^th^	60^th^
	Iron	1	16^th^	75^th^
	Magnesium	9	4^th^	47^th^
	Phosphorous	17	2^nd^	39^th^
	Potassium	12	2^nd^	53^rd^
	Selenium	4	5^th^	40^th^
	Vitamin A	15	3^rd^	18^th^
	Vitamin B1	6	5^th^	55^th^
	Vitamin B2	24	1^st^	28^th^
	Vitamin B5	10	2^nd^	41^st^
	Vitamin B6	4	5^th^	65^th^
	Vitamin B12	22	2^nd^	16^th^
	Vitamin C	3	7^th^	36^th^
	Vitamin E	1	19^th^	58^th^
	Zinc	8	3^rd^	45^th^
**Indispensable**	Cystine	7	3^rd^	39^th^
**amino acids**	Histidine	13	3^rd^	37^th^
**(bioavailability**	Leucine	17	2^nd^	35^th^
**included)**	Lysine	18	1^st^	28^th^
	Methionine	14	3^rd^	33^rd^
	Threonine	15	2^nd^	35^th^
	Tryptophan	15	3^rd^	34^th^

* *Ranked position refers to where milk appears for each nutrient on a ranked list of the 98 Food and Agriculture Organization Food Balance Sheet items. For example, milk is ranked 3^rd^ for contribution to global energy availability, after rice and wheat. In the case of nutrient density, the food items are ranked by their nutrient content per 100 g. For example, milk is ranked 54^th^ for energy density per 100 g*.

For most nutrients, this is the end of the nutrient availability calculation. However, the DELTA Model further multiplies the available nutrient by a bioavailability coefficient for protein and the indispensable amino acids to calculate the bioavailable quantity of these nutrients. The bioavailability coefficients for these nutrients in different foods are drawn from FAO data (13), and the values for milk are shown in Table 2. The bioavailability of other nutrients is not included in the DELTA Model due to insufficient data availability; this is explained further in the Discussion.

**Table 2:** Bioavailability coefficients used for protein and indispensable amino acids in the DELTA Model for milk.

**Nutrient**	**Bioavailability coefficient**
Protein	0.95
Tryptophan	0.98
Threonine	0.97
Leucine	1
Lysine	0.98
Methionine	0.92
Cystine	0.94
Histidine	1

Separately, the DELTA Model draws demographic data for the population of global regions from the United Nations (14). Nutrient reference values were taken from both the European Food Safety Authority and the FAO for the amounts of 29 modeled nutrients required daily by different age and gender groups (13, 15). The demographic and nutrient data were coupled to derive the global requirement for each nutrient. This is then appropriately divided to calculate a daily per capita target intake for each of the 29 nutrients, against which the previously calculated available quantity of these nutrients from the food system can be compared.

**Figure 1:**
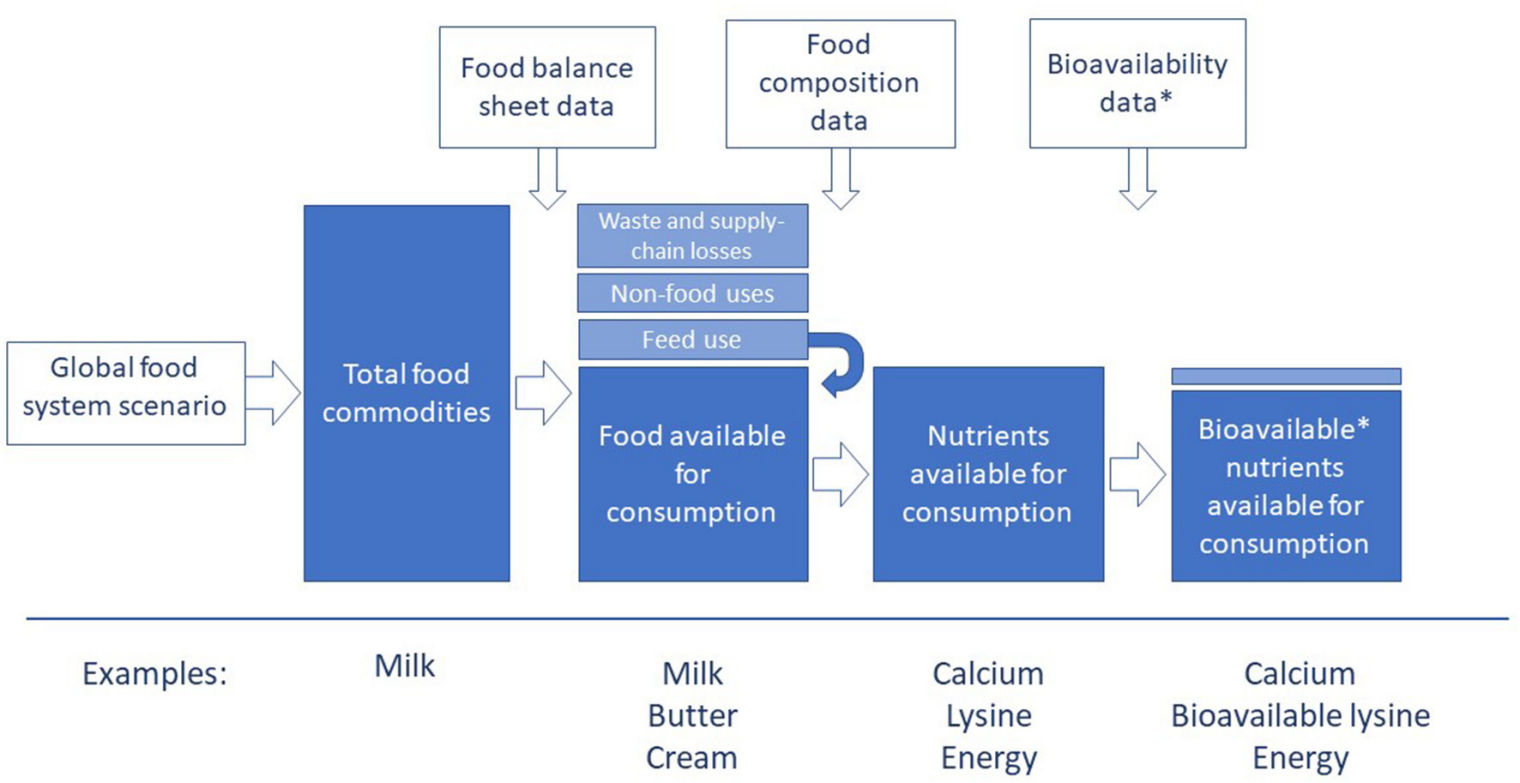
Illustration of the DELTA Model calculation process used to generate the results. See Materials and Methods and Smith et al. (10) for more detailed description. Some examples of food commodities, foods, and nutrients are shown for each stage of the calculation. *Bioavailability coefficients are applied to protein and the indispensable amino acids only.

Having used existing data to calculate the available nutrition from the global food system, the DELTA Model can also evaluate the nutritional performance of food system scenarios (Figure 1). The model user defines a global food system scenario by entering production values for 15 primary food groups, of which dairy is one. They also enter values for supply chain and in-home waste. These values are compared to the 2018 baseline data set, and the DELTA Model recalculates based on the proportional change between the newly defined scenario and the baseline.

Specific to dairy, the DELTA Model uses the global dairy production data from the FBS. This is further subdivided into more specific dairy products, such as butter, cream, buffalo milk, and so on. Here, we report the total nutritional value of milk production as an aggregated total of these products. Losses and in-home waste leading up to consumption are included, but the aggregate value is used rather than the individual nutritional contribution of each dairy product for the sake of clarity. We mention the implications of this aggregation in the discussion.

The results presented in this article pertain to the 2018 global food system.

## Results

The DELTA Model provides insight into several aspects of the global food system and global nutrition; here, we report the results pertaining to the nutrition provided by milk production in 2018. Table 1 shows the global contributions of milk to the availability of the 29 nutrients considered by the DELTA Model, as well as where milk ranked for provision and density of these nutrients on the list of the 98 food commodities used in the FBS. These results represent the relative contribution of milk to global nutrient availability in the context of all food production in 2018.

Table 1 demonstrates that milk contributed to the global availability of all but one of the nutrients considered by the DELTA Model. The most notable contributions were to calcium, protein and the indispensable amino acids, dietary fat, phosphorous, potassium and several vitamins, providing 10–49% of the global availability of these nutrients from all food sources.

Table 1 also shows that milk was one of the five most important nutrient contributors for 23 of the 29 nutrients considered by the DELTA Model. This finding was partly due to high global milk consumption, and also due to the wide range of nutrients found in milk. The high nutrient contributions of milk were coupled with low rankings for nutrient density in Table 1, with calcium the only nutrient for which milk held a top 10 place for nutrient density. This coupling is partly due to the water content of milk, and partly due to the low consumption of the most nutrient-dense foods for each nutrient. For example, seeds and spices placed higher for calcium density than milk, while tea and coffee had the greatest Vitamin B2 density, but these were relatively minor contributors to 2018 global food availability.

The DELTA Model considers the bioavailability of protein and seven indispensable amino acids using digestibility coefficients drawn from the literature (16). Table 2 shows the coefficients used by the model. These coefficients are universally above 0.9, with the lowest coefficients found for the sulfur amino acids cystine and methionine, meaning that a very high proportion of all these nutrients will be absorbed by the consumer. The sulfur amino acids are also the limiting indispensable amino acids in milk, although these amino acids are still present at high enough concentrations to make milk a complete source of these nutrients (13). The high bioavailability coefficients of milk for protein and the indispensable amino acids in Table 2 will have contributed to the high percentage contribution and ranked position of milk to the global availability of these nutrients in Table 1.

The DELTA Model splits waste into supply chain losses and in-home waste. Supply chain losses after the farm gate are considered in the FAO FBS, but in-home waste is not. The DELTA Model uses in-home waste proportions for milk of between 0.1 and 15%, taken from FAO estimates for different global regions (11). When calculated globally, DELTA found that 7% of total milk production is wasted between the farm gate and consumption. This result was less than other staple foods, such as rice (20%), wheat and products (16%), vegetables (22–27%), and the main meat items (9–10%).

By mass available for consumption, milk nutrition made up 13% of average global food availability in 2018, averaging 227 g per person per day out of a total of 1.76 kg.

## Discussion

The main findings of this research were the high contributions of milk to the global availability of several essential nutrients, in particular calcium, protein and the indispensable amino acids, dietary fat, phosphorous, potassium, and several vitamins. These results demonstrate the role that milk had in global nutrition in 2018, which is likely very similar today.

The DELTA Model has several limitations. It does not allow for detailed insight into the value of individual dairy food items to global nutrition. The FAO FBS split dairy into three items: Milk—Excluding Butter; Butter, Ghee; and Cream. The DELTA Model further subdivides the milk food items into bovine, buffalo, and sheep, but this does not include other common dairy foods, such as cheeses. The DELTA Model captures the nutrients available from milk production but not how these nutrients are consumed. In that sense, the DELTA Model returns the maximum nutrition available from milk production, after waste and losses, but some nutritional changes will occur in the downstream processing of milk into various dairy products. However, whole and skimmed milk account for around 70% of dairy food mass available for consumption, according to the more detailed FAO supply utilization accounts for processed dairy, which consider the global availability of 35 different dairy foods (9). Thus, it is likely that the DELTA Model's calculation of milk nutrition will be an indicative representation of true global nutrient availability.

The lack of granularity in the FAO data is one of its weaknesses. Other weaknesses in this data include: the omission of data from several countries; the reporting of available food rather than food eaten (addressed in the DELTA Model via the removal of inedible portions and in-home waste fractions); and the myriad differences that exist between individual food items that have been produced in different global regions. However, the FAO data are the most complete source of publicly accessible global food production and utilization data, and their annual updates will allow for future improvement in the accuracy and resolution of the DELTA Model.

Another limitation of this analysis is its global perspective, which does not allow the relative importance of milk (and different milk types) in different parts of the world. For example, 49% of milk production in India is from buffalo, compared to the global proportion of 15% (9). As buffalo milk has a slightly different recorded composition to cow's milk [(12); e.g., higher energy, calcium content; lower zinc content], this will have implications for nutrient availability from milk in India. This is compounded by differing consumption levels in different parts of the world (see later in the discussion), which will together lead to a differing dependence of regional populations on milk as a source of nutrients. The DELTA Model cannot currently capture these local differences in nutrient availability, and instead provides the global perspective.

The forecast increase in demand for protein in the future is common rhetoric in the sustainable nutrition literature (17, 18). While demand and nutritional requirement are different concepts, the current importance of milk in supplying bioavailable indispensable amino acids is clear from the DELTA Model results presented here. One of the strengths of the DELTA Model is its consideration of protein and indispensable amino acid bioavailability. It is worthwhile explaining the importance of protein quality and bioavailability with the context of several food sources. Using the Digestible Indispensable Amino Acid Score [DIAAS; (13)], milk has among the highest protein quality of common protein sources. Digestible Indispensable Amino Acid Score is the current FAO-recommended method for establishing protein quality. The calculation of the score involves consideration of indispensable amino acid digestibility in different foods, as well as the ratio of these nutrients compared to the ratio they are required in the body. Importantly, indispensable amino acids are an absolute requirement for bodily protein synthesis; once any one of the indispensable amino acids is depleted in the body, protein synthesis cannot continue, emphasizing the importance of the ratio consideration in DIAAS.

Foods with a DIAAS of >1 are considered excellent sources of protein, while scores above 0.75 are good sources, and lower scores can make no nutritional claim on protein quality. Milk protein concentrate has a DIAAS of around 1.2, compared to that of beef (0.8–1.3), soy protein isolate (0.84–0.91), pea protein concentrate (0.62–0.82), rice (0.60), and peanuts (0.43) (19–22). The high protein quality of milk, coupled with its high contribution to global indispensable amino acid supply, demonstrates the need for milk protein in meeting increasing global protein requirements, as well as increasing demand.

Bioavailability coefficients are only used for protein and the indispensable amino acids in the DELTA Model due to a lack of digestibility data for other micronutrients in most food sources, including calcium. Estimates of the bioavailability of calcium in dairy products in the literature vary widely between foods, methodologies, and subject age (23). Plant foods are the other major source of global calcium supply, and the bioavailability of this calcium is negatively affected to varying extents by plant anti-nutrients and indigestible food structures (24). Mineral bioavailability is an area that requires future research and incorporation into the model, as consideration of digestibility will almost certainly change the results reported here.

Previous results of the DELTA Model have demonstrated that calcium is a nutrient that is undersupplied by the current global food system, and likely to remain a challenge in the future (10). Calcium is often a limiting nutrient in the human diet, coupled with being one of the least wasted (10, 25). A diet low in calcium appeared 11^th^ on the list of global deaths attributable to diet and 12^th^ for disability-adjusted life years in the Global Burden of Disease study (26). Calcium deficiency can lead to a range of negative health outcomes, predominantly related to bone health [see Ilesanmi-Oyelere and Kruger (27) and Uday and Högler (28)]. The fact that milk is the most important global supplier of calcium, responsible for nearly half of the total supply, emphasizes the importance of milk in providing this nutrient. However, the DELTA Model predicted that more than a doubling of milk production would be necessary to resolve the current global calcium deficit, without considering population increases (10). Thus, it is likely that supplementation and technological advances, such as food fortification, crop biofortification, and increasing calcium bioavailability [see Sun et al. (29) and Cormick et al. (30)] will be necessary alongside milk production to supply sufficient calcium for the global population.

Despite the large contribution of milk to the global food system, its consumption is not uniform across global regions. Per capita dairy consumption varies greatly. For example, China has relatively low per capita dairy consumption (31), and calcium intakes in much of Asia are below the global average and far below the optimal level of intake for all regions (26). In contrast, the highest per capita milk and calcium consumption is seen in Europe, Australasia, and North America, above the global average, but still more than 30% below optimal intake levels (26). Local consumption data are of great importance in assessing nutritional outcomes and should complement the global results of the DELTA Model. If a nutrient is not supplied in sufficient quantities to nourish the global population, then it cannot be sufficiently supplied at the local level in all regions.

Individual consumption data is important alongside regional data. Up to 2.64% of North American adults self-report as having a dairy allergy, and just under 2% of North American children (32). Self-reported lactose intolerance has been estimated as high as 20% in North American adult populations (33). This latter study noted that perceived or real intolerance can lead to dairy avoidance, the consequences of which are possible nutrient intake deficiencies. Individuals not consuming dairy must ensure that their nutrient requirements, particularly for calcium, are sufficiently met through other sources.

The DELTA Model demonstrates the current importance of milk to global nutrition. Were milk removed from the global food system, a suitable nutritional replacement would be challenging to find. It is known that plant-based milk alternatives generally have lower protein content, amino acid bioavailability and, even when calcium-fortified to comparable levels with bovine milk, have low calcium delivery due to solubility and digestibility issues (24). Considering other milk nutrients in addition to protein and calcium increases the challenge of finding a suitable replacement. It is likely that replacing the nutritional content of milk with other foods would also require greater concomitant energy intakes, resulting in health consequences. Replacements would also be unlikely to replicate other beneficial properties of milk, such as in hydration and exercise recovery (34), and influencing the intestinal microbiome (35). In addition to the nutritional aspects of replacing milk in the global food system, replacing the economic and cultural significance of milk would be further challenges.

One must also consider the practicality of milk and dairy products as a means of delivering nutrition. Milk and dairy products are produced and consumed in many forms throughout the world, often as a staple in traditional and modern meals. This utility is coupled with a minimal requirement for processing before consumption and the ease of transporting, storing, and reconstituting dried milk. Milk also has a demonstrated place as a cost-effective means of meeting nutritional targets (36). Finally, while the environmental sustainability of milk production has been questioned, this question would be better asked from a nutritional perspective: what is the environmental footprint of milk compared to the footprint of the equivalent amount of nutrition from other sources?

The nutrients provided by milk are required throughout the lifespan. Their presence and bioavailability in milk, alongside the non-nutritional aspects of milk, makes this food a good delivery mechanism for nutrition. We foresee an increasing need for milk in nourishing a growing global population.

## Data Availability Statement

The data analyzed in this study is subject to the following licenses/restrictions: Data will be provided on request. Requests to access these datasets should be directed to Nick Smith, n.w.smith@massey.ac.nz.

## Author Contributions

WM conceived the idea for the manuscript. NS and AF were involved in the construction of DELTA version 1.2, with supervision from JH and WM. NS analyzed the data and wrote the original draft of the manuscript. All authors contributed to reviewing and editing the final version of the manuscript.

## Conflict of Interest

AF and JH are employees of Fonterra Cooperative Group Ltd. NS and WM are employees of Massey University. All authors are affiliated with the Riddet Institute, which has a strategic partnership with Fonterra Cooperative Group Ltd.

## Publisher's Note

All claims expressed in this article are solely those of the authors and do not necessarily represent those of their affiliated organizations, or those of the publisher, the editors and the reviewers. Any product that may be evaluated in this article, or claim that may be made by its manufacturer, is not guaranteed or endorsed by the publisher.

## Footnotes

^1^ www.sustainablenutritioninitiative.com
